# Supplementary material for: Problematic online gaming mediates the association between attention-deficit/hyperactivity and subsequent mental health issues in adolescents
Source: Commun Psychol. 2025 Aug 4;3:117. doi: 10.1038/s44271-025-00296-5 (PMC12322020; doi:10.1038/s44271-025-00296-5)
Supplement: Supplementary file 2 — Supplementary Information [file 44271_2025_296_MOESM2_ESM.pdf]

Supplementary Method 1 | Details on the measurement of covariates.

The model included age (months, continuous), gender (dichotomous), body mass index (continuous), IQ (continuous), household income (JPY, < 5,000,000, 5,000,000 to 9,999,999, or ≥ 10,000,000, categorical), loneliness (dichotomous, never vs. sometimes or always), physical punishment (dichotomous, never or rarely vs. sometimes, often, or always), relationships of mother, father, and friends (all continuous), neighborhood cohesion (continuous), and gender nonconforming behavior (dichotomous, not at all vs. somewhat, sometimes, or often). Per analysis, we included the baseline level of each mental health condition (continuous), time spent gaming (dichotomous, less than one hour/day vs. one hour/day or longer), and PIU (continuous) at age 12, thereby mitigating the possibility of reverse causation. Although direct information on problematic online gaming at age 12 was unavailable, the set of time spent gaming and PIU was supposed to serve as a proxy for problematic online gaming due to its overlap, and conditioning on these variables as covariates aimed to mitigate the confounding bias caused by problematic online gaming at this age. IQ was evaluated using the short form of the Wechsler Intelligence Scale for Children-Third Edition[1]. Loneliness was evaluated using a relevant item from the SMFQ[2,3]. The relationships with mother, father, and friends were evaluated using the Network of Relationships Inventory, where a higher score indicates better relationships[4]. Neighborhood cohesion was assessed using the Neighborhood Collective Efficacy scale[5], where a higher total score indicates greater neighborhood cohesion (Cronbach's α, 0.87). Gender nonconforming behavior was assessed using the Youth Self Report[6], which asked if adolescents behaved like the opposite gender. PIU was evaluated using the modified version of the Compulsive Internet Use Scale, where a higher total score indicates greater PIU (Cronbach's α, 0.87)[7].

1. Inada N, Kamio Y. Short forms of the Japanese version WISC-3 for assessment of children with autism spectrum disorders. *Jpn J Child Adolesc Psychiatr (Japanese)*. 2010;51:11-19.
2. Angold A, Costello EJ, Messer SC, Pickles A. Development of a short questionnaire for use in epidemiological studies of depression in children and adolescents. *Int J Methods Psychiatr Res*. 1995;5(4):237-249.
3. Sharp C, Goodyer IM, Croudace TJ. The Short Mood and Feelings Questionnaire (SMFQ): a unidimensional item response theory and categorical data factor analysis of self-report ratings from a community sample of 7-through 11-year-old children. *J Abnorm Child Psychol*. 2006;34(3):379-391.
4. Furman W, Buhrmester D. The Network of Relationships Inventory: Behavioral Systems Version. *Int J Behav Dev*. 2009;33(5):470-478.
5. Nakanishi M, Yamasaki S, Ando S, et al. Neighborhood Social Cohesion and Dementia-Related Stigma Among Mothers of Adolescents in the Pre- and Current COVID-19 Period: An Observational Study Using Population-Based Cohort Data. *J Alzheimers Dis*. 2022;88(2):493-502.
6. Achenbach TM, Rescorla L. *Manual for the ASEBA School-Age Forms & Profiles*. ASEBA; 2001.
7. Meerkerk GJ, Van Den Eijnden RJJM, Vermulst AA, Garretsen HFL. The Compulsive Internet Use Scale (CIUS): some psychometric properties. *Cyberpsychol Behav*. 2009;12(1):1-6.

Supplementary Method 2 | The calculations for the relative excess risk due to interaction (RERI) as an additive scale and the ratio of risk ratios (RRR) as a multiplicative scale.

$$RERI = RR_{11} - RR_{01} - RR_{10} + 1$$

$$RRR = RR_{11}/(RR_{01}*RR_{10})$$

RR_{11} is the risk ratio for mental health issues when both the high degree of problematic online gaming and the female gender are present.
RR_{01} is the risk ratio for mental health issues when the high degree of problematic online gaming is present, but the female gender is not present.
RR_{10} is the risk ratio for mental health issues when the high degree of problematic online gaming is not present, but the female gender is present.

Supplementary Fig. 1 | Association between problematic online gaming at age 14 and mental health at age 16.

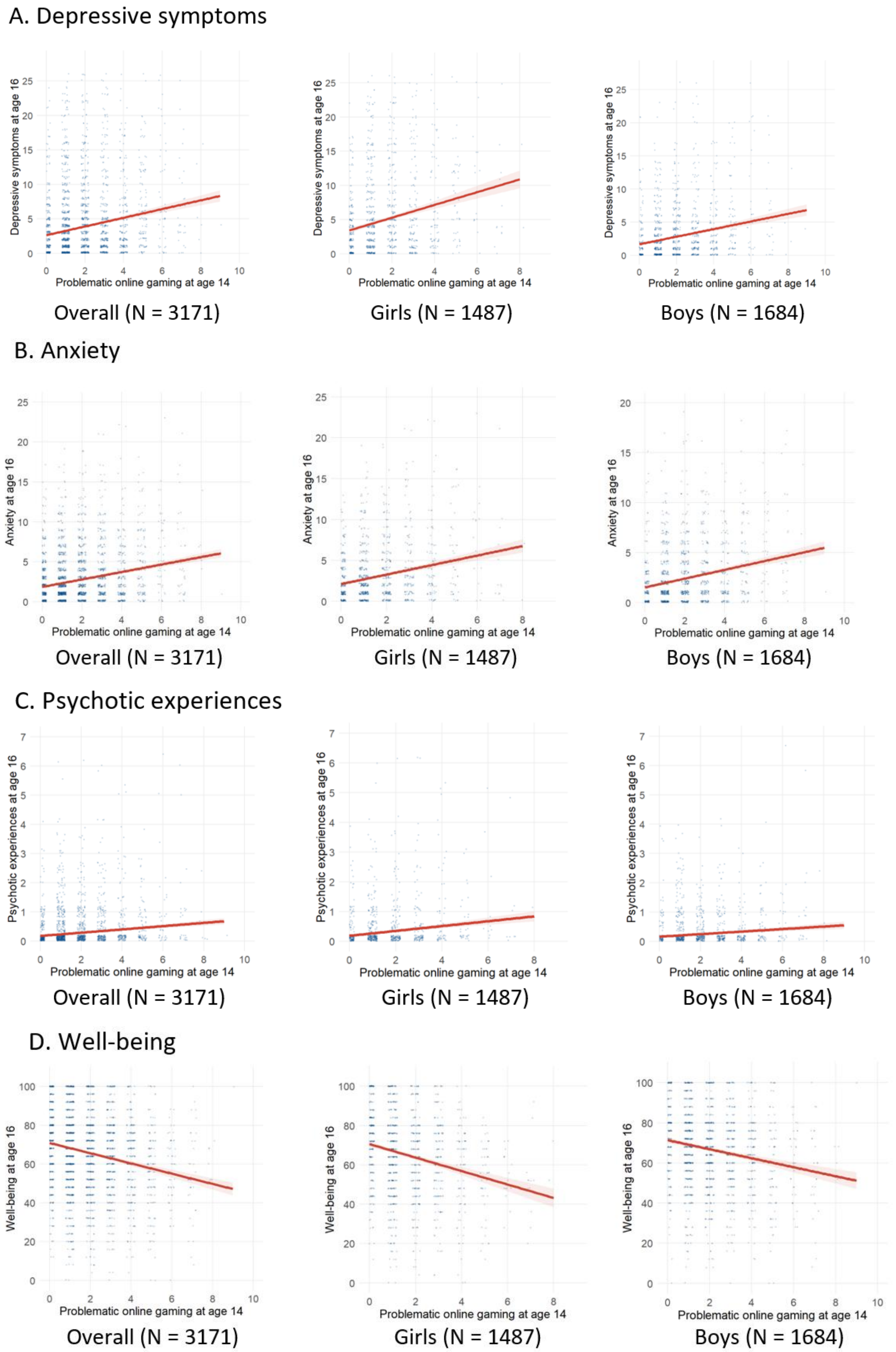

Supplementary Fig. 2 | Association between attention-deficit/hyperactivity at age 12 and problematic online gaming at age 14.

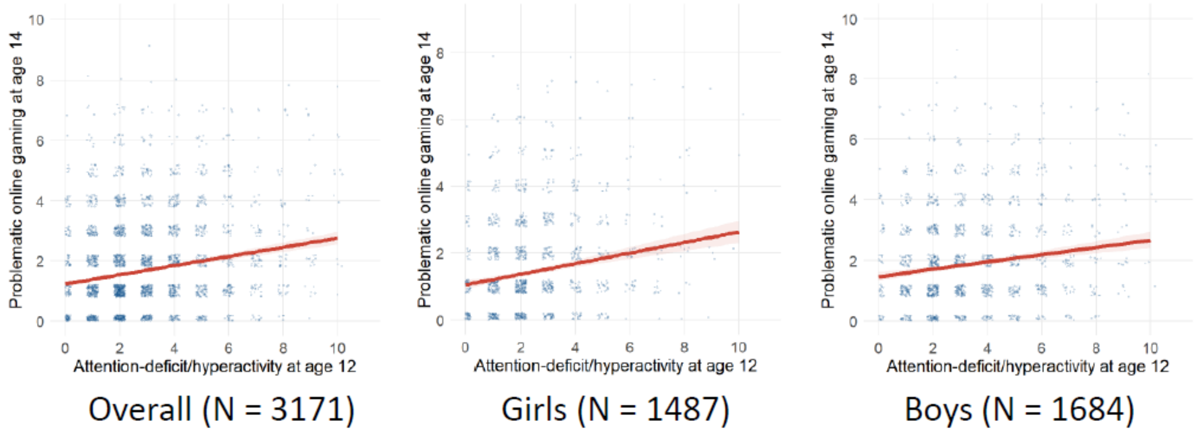

Supplementary Fig. 3 | Sensitivity analysis for proportion mediated: excluding loneliness from the model.

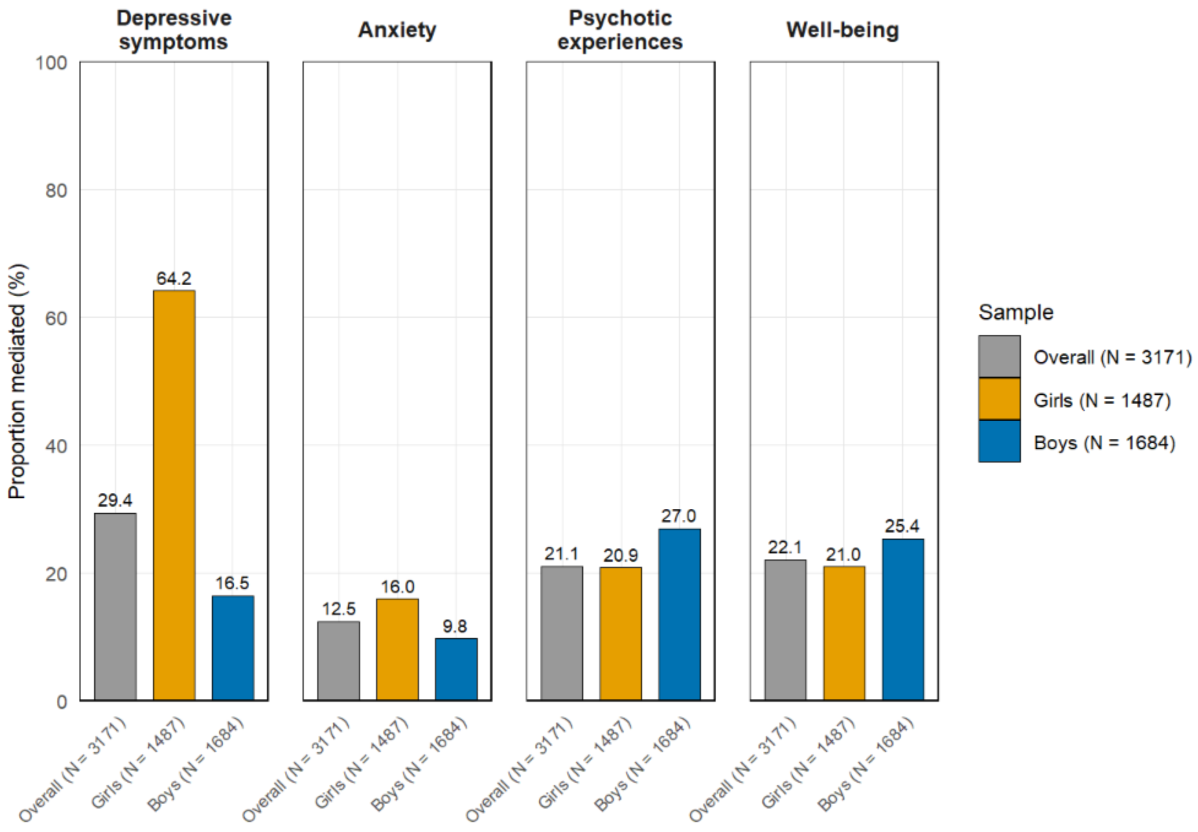

Supplementary Table 1 | Problematic online gaming measurements.

Items
1. Changes in gaming methods, such as increasing time, paying money, and switching to more difficult games to achieve the desired excitement (satisfaction).
2. Is restless or irritable when attempting to cut down or stop online gaming.
3. Has made repeated unsuccessful efforts to control, cut back, or stop online gaming.
4. Is often preoccupied with online gaming (e.g., having persistent thoughts of reliving past online gaming experiences, gathering the latest game information, making an action plan for the next online game, constantly thinking about how to get time to play online games, and how to get money to pay for games).
5. Often plays online games when feeling distressed (e.g., helpless, guilty, anxious, or depressed).
6. When not getting what they want in an online game (e.g., they did not get the item they want or did not clear the event), often returning on another day to accomplish this.
7. Lies to conceal the extent of involvement with online gaming.
8. Has jeopardized or impaired school performance, schoolwork, or a significant relationship because of online gaming.
9. Relies on others to provide relief from desperate situations caused by online gaming (e.g., borrowing money).

Supplementary Table 2 | The Adolescent Psychotic-like Symptom Screener.

Questionnaire items	Abbreviation
1. Some people believe that their thoughts can be read by another person. Have other people ever read your mind?	Mind reading
2. Have you ever had messages sent just to you through TV or radio?	TV/radio
3. Have you ever thought that people are following or spying on you?	Spying
4. Have you ever heard voices or sounds that no one else can hear?	Auditory hallucinations
5. Have you ever felt you were under the control of some special power?	Controlled
6. Have you ever seen things that other people could not see?	Visual hallucinations
7. Have you ever felt like you had extraspecial powers?	Grandiosity

Supplementary Table 3 | Gender-specific scores for each measure.

Measure	Age	Boys<br>Mean (SD)	Girls<br>Mean (SD)
Attention-deficit/hyperactivity	12	3.12 (2.16)	2.26 (1.86)
Problematic online gaming	14	1.98 (1.71)	1.52 (1.63)
Depressive symptoms	16	2.72 (4.18)	4.85 (5.93)
Anxiety	16	2.26 (3.05)	2.96 (3.70)
Psychotic experiences	16	0.24 (0.66)	0.27 (0.67)
Well-being	16	67.4 (20.2)	65.5 (21.8)

SD, standard deviation

Supplementary Table 4 | Association between problematic online gaming at age 14 and mental health at age 16: analyzing via the doubly robust estimator.

Samples and outcomes	Groups	RD, % [95% CI]	RR [95% CI]
Overall			
Incident depression	Low degree of problematic online gaming (0–3 points)	Reference	Reference
	High degree of problematic online gaming (4–9 points)	7.8 [3.0, 13.1]	1.62 [1.25, 2.05]
Incident anxiety	Low degree of problematic online gaming (0–3 points)	Reference	Reference
	High degree of problematic online gaming (4–9 points)	5.7 [2.7, 8.7]	1.98 [1.45, 2.67]
Incident psychotic experiences	Low degree of problematic online gaming (0–3 points)	Reference	Reference
	High degree of problematic online gaming (4–9 points)	5.9 [2.3, 10.8]	1.72 [1.30, 2.47]
Diminished well-being	Low degree of problematic online gaming (0–3 points)	Reference	Reference
	High degree of problematic online gaming (4–9 points)	9.6 [5.1, 14.3]	1.54 [1.27, 1.84]
Girls			
Incident depression	Low degree of problematic online gaming (0–3 points)	Reference	Reference
	High degree of problematic online gaming (4–9 points)	13.9 [5.9, 24.4]	1.80 [1.32, 2.46]
Incident anxiety	Low degree of problematic online gaming (0–3 points)	Reference	Reference
	High degree of problematic online gaming (4–9 points)	5.9 [0.3, 12.2]	1.78 [1.04, 2.68]
Incident psychotic experiences	Low degree of problematic online gaming (0–3 points)	Reference	Reference
	High degree of problematic online gaming (4–9 points)	7.3 [2.0, 15.9]	1.84 [1.22, 2.99]
Diminished well-being	Low degree of problematic online gaming (0–3 points)	Reference	Reference
	High degree of problematic online gaming (4–9 points)	13.0 [3.7, 22.4]	1.67 [1.18, 2.17]
Boys			
Incident depression	Low degree of problematic online gaming (0–3 points)	Reference	Reference
	High degree of problematic online gaming (4–9 points)	3.6 [-0.9, 8.6]	1.42 [0.90, 2.11]
Incident anxiety	Low degree of problematic online gaming (0–3 points)	Reference	Reference
	High degree of problematic online gaming (4–9 points)	5.8 [2.1, 8.8]	2.32 [1.43, 3.34]
Incident psychotic experiences	Low degree of problematic online gaming (0–3 points)	Reference	Reference
	High degree of problematic online gaming (4–9 points)	4.5 [0.5, 9.7]	1.60 [1.01, 2.37]

Diminished well-being	Low degree of problematic online gaming (0–3 points)	Reference	Reference
	High degree of problematic online gaming (4–9 points)	6.9 [1.9, 12.1]	1.43 [1.11, 1.85]

RD, risk difference; RR, risk ratio; CI, confidence interval
Adolescents who already had each mental health outcome at age 12 were excluded per the analysis.
Missing data were handled using random forest imputation.
The model included age, gender, body mass index, IQ, household income, loneliness, physical punishment, relationships with mother, father, and friends, neighborhood cohesion, gender nonconformity, attention-deficit/hyperactivity, and each mental health variable at age 12.
CIs were obtained via percentiles of 200 bootstrap samples.

Supplementary Table 5 | Additive and multiplicative interactions between problematic online gaming and gender.

Outcomes	RERI [95% CI]	RRR [95% CI]
Incident depression	1.21 [0.11, 2.55]	1.26 [0.73, 2.14]
Incident anxiety	0.04 [-1.72, 1.75]	0.76 [0.37, 1.58]
Incident psychotic experiences	0.37 [-0.57, 1.55]	1.15 [0.65, 2.10]
Diminished well-being	0.39 [-0.35, 1.08]	1.16 [0.74, 1.69]

CI, confidence interval; RERI, relative excess risk due to interaction; RRR, ratio of risk ratio
The CIs for RERI and RRR were derived from percentiles based on 200 bootstrap samples.
We fitted the following Poisson regression model:
log{P(Mental health issues = 1| Problematic online gaming, Gender, Covariates)}
= b_0 + b_1 Gender + b_2 Problematic online gaming_high + b_3 Problematic online gaming_high * Gender + b_4 Covariates

Supplementary Table 6 | Sensitivity analysis: analyzing problematic online gaming as a dichotomous variable.

	Depressive symptoms		Anxiety		Psychotic experiences		Well-being	
Samples and groups	*β* [95% CI]	*P*	*β* [95% CI]	*P*	*β* [95% CI]	*P*	*β* [95% CI]	*P*
Overall (N = 3171)								
Low degree of problematic online gaming (0–3 points)	Reference		Reference		Reference		Reference	
High degree of problematic online gaming (4–9 points)	1.54 [1.04, 2.03]	< 0.001	0.83 [0.55, 1.11]	< 0.001	0.17 [0.11, 0.24]	< 0.001	-6.14 [-8.16, -4.11]	< 0.001
Girls (N = 1487)								
Low degree of problematic online gaming (0–3 points)	Reference		Reference		Reference		Reference	
High degree of problematic online gaming (4–9 points)	1.90 [0.97, 2.82]	< 0.001	0.98 [0.50, 1.46]	< 0.001	0.26 [0.13, 0.38]	< 0.001	-8.46 [-11.84, -5.08]	< 0.001
Boys (N = 1684)								
Low degree of problematic online gaming (0–3 points)	Reference		Reference		Reference		Reference	
High degree of problematic online gaming (4–9 points)	1.36 [0.83, 1.90]	< 0.001	0.71 [0.39, 1.04]	< 0.001	0.12 [0.04, 0.20]	0.002	-4.68 [-7.18, -2.18]	< 0.01

Missing data were handled using random forest imputation.
The model included age, gender, body mass index, IQ, household income, loneliness, physical punishment, relationships with mother, father, and friends, neighborhood cohesion, gender nonconformity, attention-deficit/hyperactivity, and each mental health variable at age 12.

Supplementary Table 7 | Sensitivity analysis for the association between problematic online gaming at age 14 and mental health at age 16: analyzing mental health as dichotomous variables.

Samples and outcomes	RR (per 1 SD) [95% CI]	*P*
Overall		
Incident depression	1.30 [1.18, 1.43]	< 0.001
Incident anxiety	1.37 [1.21, 1.55]	< 0.001
Incident psychotic experiences	1.34 [1.20, 1.50]	< 0.001
Diminished well-being	1.23 [1.13, 1.33]	< 0.001
Girls		
Incident depression	1.29 [1.14, 1.45]	< 0.001
Incident anxiety	1.40 [1.16, 1.67]	< 0.001
Incident psychotic experiences	1.34 [1.14, 1.57]	< 0.001
Diminished well-being	1.23 [1.11, 1.37]	< 0.001
Boys		
Incident depression	1.31 [1.12, 1.53]	< 0.001
Incident anxiety	1.41 [1.18, 1.69]	< 0.001
Incident psychotic experiences	1.33 [1.13, 1.55]	< 0.001
Diminished well-being	1.20 [1.07, 1.34]	0.001

RR, risk ratio; SD, standard deviation; CI, confidence interval
RR is reported per one SD increase in the exposure.
Adolescents who already had each mental health outcome at age 12 were excluded per the analysis.
Missing data were handled using random forest imputation.
The model included age, gender, body mass index, IQ, household income, loneliness, physical punishment, relationships with mother, father, and friends, neighborhood cohesion, gender nonconformity, attention-deficit/hyperactivity, and each mental health variable at age 12.

Supplementary Table 8 | Sensitivity analysis for the association between problematic online gaming at age 14 and mental health at age 16: robustness to unmeasured confounding of the observed associations.

Samples	E-value	Incident depression	Incident anxiety	Incident psychotic experiences	Diminished well-being
Overall	Point estimate	2.62	3.37	2.82	2.45
	Limit of CI	1.81	2.26	1.92	1.86
Girls	Point estimate	3.00	2.95	3.08	2.62
	Limit of CI	1.97	1.24	1.74	1.64
Boys	Point estimate	2.19	4.07	2.58	2.24
	Limit of CI	1.00	2.21	1.11	1.46

CI, confidence interval
E-values for point estimates are the minimum strength of association on the risk ratio that unmeasured confounding would need to have above and beyond the adjusted covariates to explain away the estimates.
E-values for limit of CI are the minimum strength of association on the risk ratio that unmeasured confounding would need to have above and beyond the adjusted covariates to shift the 95% CI and include the null value.

Supplementary Table 9 | Sensitivity analysis for the association between problematic online gaming at age 14 and mental health at age 16: excluding loneliness from the model.

Samples and outcomes	*β* (per 1 SD) [95% CI]	*P*
Overall (N = 3171)		
Depressive symptoms	0.91 [0.73, 1.08]	< 0.001
Anxiety	0.45 [0.35, 0.54]	< 0.001
Psychotic experiences	0.09 [0.06, 0.11]	< 0.001
Well-being	-3.27 [-3.98, -2.56]	< 0.001
Girls (N = 1487)		
Depressive symptoms	1.08 [0.79, 1.37]	< 0.001
Anxiety	0.45 [0.30, 0.60]	< 0.001
Psychotic experiences	0.11 [0.07, 0.15]	< 0.001
Well-being	-3.86 [-4.93, -2.80]	< 0.001
Boys (N = 1684)		
Depressive symptoms	0.76 [0.56, 0.95]	< 0.001
Anxiety	0.42 [0.30, 0.54]	< 0.001
Psychotic experiences	0.07 [0.04, 0.10]	< 0.001
Well-being	-2.72 [-3.65, -1.78]	< 0.001

SD, standard deviation; CI, confidence interval
β is reported per one SD increase in the exposure; its magnitude depends on each outcome's scale and is not comparable across outcomes.
Missing data were handled using random forest imputation.
The model included age, gender, body mass index, IQ, household income, loneliness, physical punishment, relationships with mother, father, and friends, neighborhood cohesion, gender nonconformity, attention-deficit/hyperactivity, and each mental health variable at age 12.

Supplementary Table 10 | Association of attention-deficit/hyperactivity at age 12 with problematic online gaming at age 14.

Samples	Model 1		Model 2		Model 3	
	β (per 1 SD) [95% CI]	*P*	*β* (per 1 SD) [95% CI]	*P*	*β* (per 1 SD) [95% CI]	*P*
Overall (N = 3,171)	0.32 [0.26, 0.37]	< 0.001	0.28 [0.22, 0.34]	< 0.001	0.18 [0.12, 0.24]	< 0.001
Girls (N = 1,487)	0.29 [0.22, 0.37]	< 0.001	0.29 [0.21, 0.37]	< 0.001	0.18 [0.10, 0.26]	< 0.001
Boys (N = 1,684)	0.26 [0.18, 0.34]	< 0.001	0.26 [0.18, 0.34]	< 0.001	0.18 [0.10, 0.27]	< 0.001

SD, standard deviation; CI, confidence interval
β is reported per one SD increase in the exposure; its magnitude depends on each outcome's scale and is not comparable across outcomes.
Missing data were handled using random forest imputation.
Model 1 was unadjusted.
Model 2 adjusted for age, gender, body mass index, IQ, and household income at age 12.
Model 3 adjusted for the variables included in Model 2, loneliness, physical punishment, relationships with mother, father, and friends, neighborhood cohesion, gender nonconformity, time spent gaming, and problematic internet use at age 12.

Supplementary Table 11 | Sensitivity analysis for the association of attention-deficit/hyperactivity at age 12 and problematic online gaming at age 14: analyzing problematic online gaming as a dichotomous variable.

Samples	RR (per 1 SD) [95% CI]	*P*
Overall (N = 3171)	1.21 [1.10, 1.32]	< 0.001
Girls (N = 1487)	1.27 [1.10, 1.46]	0.001
Boys (N = 1684)	1.18 [1.05, 1.33]	0.004

SD, standard deviation; CI, confidence interval
RR is reported per one SD increase in the exposure.
Missing data were handled using random forest imputation.
The model included age, gender, body mass index, IQ, household income, loneliness, physical punishment, relationships with mother, father, and friends, neighborhood cohesion, gender nonconformity, attention-deficit/hyperactivity, and each mental health variable at age 12.

Supplementary Table 12 | Sensitivity analysis for the mediation effect: excluding loneliness from the model.

Samples and outcomes	Total effect β (per 1 SD) [95% CI]	Pure direct effect β (per 1 SD) [95% CI]	Total indirect effect β (per 1 SD) [95% CI]	*P* for total indirect effect
Overall (N = 3171)				
Depressive symptoms	0.26 [0.07, 0.45]	0.18 [0.00, 0.37]	0.08 [0.04, 0.11]	< 0.001
Anxiety	0.33 [0.22, 0.43]	0.29 [0.18, 0.39]	0.04 [0.02, 0.06]	< 0.001
Psychotic experiences	0.06 [0.03, 0.08]	0.04 [0.02, 0.07]	0.01 [0.01, 0.02]	< 0.001
Well-being (reverse scored)	1.30 [0.53, 2.06]	1.01 [0.24, 1.78]	0.29 [0.15, 0.42]	< 0.001
Girls (N = 1487)				
Depressive symptoms	0.16 [-0.15, 0.47]	0.06 [-0.25, 0.37]	0.10 [0.04, 0.17]	0.001
Anxiety	0.29 [0.13, 0.46]	0.26 [0.09, 0.42]	0.05 [0.02, 0.08]	0.004
Psychotic experiences	0.08 [0.04, 0.12]	0.06 [0.02, 0.10]	0.02 [0.01, 0.03]	< 0.001
Well-being (reverse scored)	1.67 [0.54, 2.80]	1.32 [0.19, 2.45]	0.35 [0.13, 0.57]	0.002
Boys (N = 1684)				
Depressive symptoms	0.38 [0.16, 0.59]	0.31 [0.10, 0.53]	0.06 [0.02, 0.11]	0.005
Anxiety	0.36 [0.23, 0.49]	0.32 [0.19, 0.46]	0.04 [0.01, 0.06]	0.005
Psychotic experiences	0.03 [0.00, 0.07]	0.03 [-0.01, 0.06]	0.01 [0.003, 0.02]	0.002
Well-being (reverse scored)	0.99 [-0.01, 1.99]	0.74 [-0.27, 1.74]	0.25 [0.08, 0.42]	0.004

SD, standard deviation; CI, confidence interval
See the methods section for a detailed interpretation of β.
The analysis accounted for the interaction of attention-deficit/hyperactivity and problematic online gaming.
Missing data were handled using random forest imputation.
The model adjusted for age, gender, body mass index, IQ, household income, loneliness, physical punishment, relationships with mother, father, and friends, neighborhood cohesion, gender nonconformity, time spent gaming, problematic internet use, and each mental health variable at age 12.
CIs were computed using the delta method.
